# The Long Shadow of Early HCMV–HIV Coinfection: Epidemiology, Pathogenesis, and Immune Consequences

**DOI:** 10.3390/children13020236

**Published:** 2026-02-07

**Authors:** Camilla Albano, Francesca Gugliesi, Greta Bajetto, Beatrice Braga, Valentina Dell’Oste, Gloria Griffante, Selina Pasquero

**Affiliations:** Department of Public Health and Pediatric Sciences, Medical School, University of Turin, 10124 Turin, Italy

**Keywords:** HCMV, HIV, coinfection, congenital infections, immunosenescence, cellular senescence, premature aging

## Abstract

Human cytomegalovirus (HCMV) and Human Immunodeficiency Virus (HIV) are two pathogens known to have dramatic consequences when contracted early in life. In addition to having a significant impact when acquired individually, these two viruses are known to frequently cause coinfections. Indeed, also in the modern era, HCMV remains one of the most prevalent coinfections in newborns of mothers living with HIV, including both HIV-positive children regardless of their immune status, and those exposed to HIV but uninfected (HEU). In children with HIV infection, HCMV coinfection has historically been associated with AIDS-defining disease, high mortality, and prolonged, elevated HCMV viral load. Although timely administration of antiretroviral therapy prevents immunodeficiency in people living with HIV and thus reduces the incidence of full-blown HCMV disease in cases of coinfection, emerging data suggest that HCMV-induced immune activation and aging persist, potentially contributing to long-term, non-AIDS-related comorbidities. Growing evidence indicates that also HCMV amplifies HIV susceptibility, disease progression, and immune dysregulation through multiple synergistic mechanisms. Moreover, congenital and early postnatal HCMV infections occur at significantly higher rates in HEU newborns than in HIV-unexposed children and are associated with worse clinical outcomes, particularly when HCMV viral loads are high. This review summarizes current knowledge on the epidemiology, clinical impact, and immunopathogenetic interactions of early HCMV–HIV coinfection in pediatric populations. By integrating recent findings with historical evidence, it highlights critical mechanistic and epidemiological gaps that warrant further investigation.

## 1. Introduction

Human cytomegalovirus (HCMV) and Human Immunodeficiency Virus (HIV) are two globally prevalent pathogens that pose substantial health risks during pregnancy, infancy, and early childhood. Both viruses establish lifelong infections, exhibit complex interactions with the host immune system, and disproportionately affect individuals with immature or compromised immunity, making their intersection particularly relevant in maternal–child health.

HCMV is a ubiquitous β-herpesvirus that infects 56% to 94% of the adult human population worldwide [1]. Its success as a human pathogen reflects its broad cellular tropism, capacity for systemic dissemination, prolonged shedding in bodily fluids, including saliva, urine, breast milk, and genital secretions, and lifelong latency within cells of the myeloid lineage, with the potential for periodic reactivation [2]. While primary HCMV infection is typically asymptomatic in immunocompetent adults, fetuses and young infants, whose immune systems are immature or impaired, are particularly vulnerable to severe disease.

Congenital HCMV (cCMV) infection is the most common congenital viral infection globally, affecting approximately 0.2–2% of newborns, with prevalence varying by geographic region, ethnicity, and socioeconomic status [3]. In its most severe cases, cCMV infection can result in fetal demise. Among live-born infants, approximately 10–15% are symptomatic at birth, presenting with manifestations such as intrauterine growth restriction, jaundice, hepatosplenomegaly, petechial rash, retinitis, seizures, microcephaly, and other neurological abnormalities [4]. Importantly, even infants who are asymptomatic at birth remain at risk for long-term sequelae, most notably sensorineural hearing loss, which represents the most common permanent disability associated with cCMV and the leading non-genetic cause of childhood hearing impairment [5].

In addition to transplacental transmission, infants may acquire HCMV intrapartum through exposure to cervicovaginal secretions or postnatally, most commonly via breast milk [6]. Although postnatal HCMV infection is generally mild or asymptomatic in healthy term infants, it can cause clinically significant disease in preterm or immunocompromised children. Notably, HCMV infection in infants born to mothers living with HIV has been associated with increased HIV transmission and more rapid progression of pediatric HIV disease [7], highlighting biologically and clinically important interactions between these two viruses early in life.

HIV is a lentivirus of the Retroviridae family characterized by its ability to reverse-transcribe its RNA genome into DNA and integrate into the host cell genome, thereby establishing lifelong infection. HIV primarily targets CD4^+^ T lymphocytes but also infects monocytes, macrophages, and glial cells [8]. Progressive depletion and dysfunction of CD4^+^ T cells ultimately lead to acquired immunodeficiency syndrome (AIDS), marked by profound immune suppression and heightened susceptibility to opportunistic infections.

While sexual transmission and exposure to infected blood account for the majority of HIV infections worldwide, mother-to-child transmission (MTCT) remains the principal route of pediatric HIV acquisition [9]. The clinical course of pediatric HIV infection is highly variable and influenced by disease stage, timing of diagnosis, and access to appropriate care [10]. In the absence of treatment, pediatric HIV infection is associated with extremely high mortality; in resource-limited settings, mortality rates reached approximately 40% within the first year of life and 60% by 3–5 years of age [11]. Although early initiation of antiretroviral therapy (ART) has dramatically improved survival and quality of life, ART suppresses viral replication without eliminating the virus. As a result, individuals infected early in life face lifelong HIV infection, persistent immune dysregulation, and prolonged exposure to antiretroviral therapy.

Together, the overlapping epidemiology, shared transmission routes, and intersecting immunopathogenic mechanisms of HCMV and HIV create a unique and clinically significant context in which coinfection may shape disease outcomes across the life course. This review examines current evidence on the impact of HCMV and HIV coinfection, with particular emphasis on maternal–child transmission, pediatric outcomes, and the cooperative mechanisms through which these two viruses influence pathogenesis.

## 2. Classification, Frequency, and Impacts of HCMV–HIV Coinfection

Understanding the frequency and directionality of HCMV–HIV coinfection requires careful consideration of the different transmission routes and timing of acquisition of each virus. Both HIV and HCMV can be acquired in utero, during delivery, or postnatally (Table 1); however, the relative contribution of these routes differs substantially between the two pathogens. cCMV infection represents the most common congenital infection worldwide, whereas in the era of ART, MTCT of HIV occurs predominantly during childbirth or breastfeeding rather than during pregnancy [12,13,14,15].

Accurate determination of the timing of viral acquisition is essential for interpreting epidemiological associations between HIV and HCMV, particularly because the immunological and clinical consequences of coinfection are likely to be highly time-dependent. Current diagnostic criteria (Table 1) allow for more precise temporal classification of infection compared with studies conducted before 2000 [7,16,17], when standardized criteria were not consistently applied. According to current definitions, HCMV infection is classified as congenital when viral DNA is detected by PCR or when the virus is isolated within the first 21 days of life [3,15]. In contrast, HIV infection is considered in utero when PCR positivity is documented within the first 48–72 h after birth; positivity between day 3 and the first month of life is indicative of intrapartum transmission, whereas later detection reflects postnatal acquisition, most commonly through breastfeeding [12]. These criteria have significantly improved the ability to examine the co-transmission pattern.

In the following paragraphs, we review studies addressing whether infants living with HIV have an increased risk of acquiring HCMV, whether HIV exposure alone, among HIV-exposed but uninfected (HEU) infants, confers heightened susceptibility to HCMV infection, and whether HCMV infection may, in turn, elevate the risk of MTCT of HIV.

### 2.1. Frequency of cCMV Infection on HIV Exposure

Numerous studies have investigated whether infants exposed to HIV in utero have an increased risk of acquiring cCMV infection [7,16,18,19,20,21,22,23]. Interpretation of early studies, however, is complicated by limitations in diagnostic resolution before 2000, when the timing of infant HIV infection could not be reliably distinguished between in utero, intrapartum, and postnatal acquisition, thereby obscuring temporal relationships between HIV exposure and HCMV transmission [17,24]. More recent studies employing standardized molecular diagnostics have enabled more accurate classification of infection timing and clearer assessment of co-transmission patterns.

An early US study, conducted between 1988 and 1995 in a cohort of 154 newborns, showed a significantly higher prevalence of cCMV infection among infants living with HIV compared to HEU infants (21% vs. 3.8%; *p* = 0.008) [23]. Concordant results emerged in the French Perinatal Cohort study, which assessed 4797 HIV-exposed newborns using urine culture within the first 10 days of life and found cCMV infection to be substantially more frequent among HIV-infected infants than among HEU infants (10.3% vs. 2.2%; *p* < 0.001) [22]. Higher prevalences of cCMV among newborns living with HIV have also been reported in smaller studies conducted in Kenya, Thailand, and Malawi [19,20,21]. Although some studies did not identify statistically significant differences by HIV status, these analyses were often underpowered due to small numbers of HIV-infected newborns, limiting statistical power [17,18,24].

### 2.2. Frequency of cCMV Infection on HIV Exposure in the Era of ART

More recent data from the ART era suggest a changing epidemiological landscape. An observational, retrospective study conducted at Hospital Universitario 12 de Octubre in Madrid between 2000 and 2017 reported lower rates of cCMV among HEU infants compared with the pre-ART era, yet still higher than those observed in the general population [25]. Consistently, a large multicenter study confirmed persistently high rates of cCMV and elevated urinary HCMV viral loads among HEU infants, even in the context of widespread ART. Notably, in utero HIV infection emerged as a major risk factor for cCMV, particularly among infants born to mothers who did not receive combination antiretroviral therapy during pregnancy [26].

Beyond epidemiological associations, recent genomic analyses have provided mechanistic insight into cCMV transmission in the context of maternal HIV infection. Given that HIV-associated immunosuppression facilitates infection with multiple HCMV strains, Pang et al. used viral genome sequencing to characterize HCMV transmission in HIV-positive mother–infant pairs [27]. Despite the presence of multiple HCMV genotypes in maternal compartments, including cervix, blood, and breast milk, congenitally infected infants typically acquired a single dominant viral strain, consistent with a stringent transmission bottleneck. The transmitted strain was genetically most similar to the virus detected in the maternal cervix, implicating this compartment as a principal source of intrauterine HCMV transmission. Moreover, congenitally transmitted strains frequently harbored genetic variants associated with cellular tropism and immune evasion, suggesting selective pressures that favor specific HCMV genotypes capable of crossing the placental barrier.

### 2.3. Frequency of HIV Exposure on Postnatal HCMV Acquisition

Regarding postnatal HCMV infection, most studies show higher rates in children living with HIV compared to HEU children [7,16,17,19,24]. The only study that did not find significant differences was that of Chandwani et al. Still, the *p* value was close to significance, and considering all HCMV infections (congenital + postnatal), the overall rate was still higher in children living with HIV (30% vs. 17%; *p* = 0.010) [17].

Recent findings confirm this trend: an analysis of post-ART cohorts reported a high prevalence of HCMV viremia at 6 months in infants born to mothers living with HIV, despite prolonged maternal ART. This indicates that in HEU infants, early HCMV acquisition and its replication or persistence remain significant even during the ART era and are linked to increased immune activation, which could potentially influence susceptibility to HIV transmission [28]. In a 2016 Zimbabwean study, HCMV infection and inflammation were examined in 231 HEU infants and 100 HIV-unexposed 6-week-old infants. Interestingly, the infection rates did not differ between the groups, with high HCMV prevalence in both (81.4% vs. 74.0%; *p* = 0.14). However, HEU infants exhibited significantly higher HCMV viral loads (*p* = 0.005). This was also associated with increased inflammation, suggesting that maternal HIV exposure influences the immune response and the severity of HCMV infection. These data highlight that future research should focus not only on the presence or absence of infection but also on its extent, measured by viral load, and the related inflammatory response [29].

With respect to postnatal infection, it is important to note that infants with cCMV can acquire additional viral strains after birth. In a longitudinal postnatal study, Pang et al. demonstrated that all infants, including those congenitally infected, acquired new HCMV strains postnatally, predominantly through breastfeeding [27]. Breast milk from mothers living with HIV contained multiple HCMV genotypes, frequently distinct from the congenital strain, and infants rapidly developed superinfections with genetically divergent viruses. These findings indicate that, in the context of maternal HIV infection, postnatal HCMV transmission is a highly dynamic process characterized by repeated exposure and sequential acquisition of diverse strains, thereby expanding viral diversity in infants and sustaining immune activation during the early months of life.

### 2.4. Impact of HCMV Infection on HIV Mother-to-Child Transmission

Fewer studies have examined the reciprocal relationship, namely, whether HCMV infection increases susceptibility to HIV MTCT. In a study from Thailand, Khamduang and colleagues reported that cCMV infection, defined by the presence of HCMV-specific IgM in cord blood or detection of viral DNA within the first 10 days of life, was associated with an increased risk of intrapartum HIV transmission. In contrast, perinatal HCMV infection showed no such association [19]. In contrast, a study conducted in Malawi by Chang et al. [21] found that HCMV infection detected at 6 months of age, reflecting postnatal acquisition during breastfeeding, was not significantly associated with HIV transmission via breast milk, although a marginal association was observed with the composite outcome of “HIV infection or infant death”.

Collectively, these findings suggest that the timing of HCMV infection is a critical determinant of its impact on HIV MTCT: cCMV appears to increase susceptibility to HIV transmission, whereas postnatal HCMV infection exerts a more limited effect.

## 3. Morbidity and Mortality in HCMV–HIV Co-Infected Infants

### 3.1. HCMV-Related Morbidity in Adults Living with HIV

Before the widespread availability of ART, HCMV was among the most frequent and severe opportunistic infections in people living with HIV [30]. It commonly manifested as an AIDS-defining condition, often referred to as AIDS-related disease, reflecting clinical events directly attributable to profound immunodeficiency, typically in the setting of very low CD4^+^ T-cell counts (Table 2). Despite major advances in HIV care, HCMV remains the most prevalent viral coinfection in immunocompromised individuals, particularly among those without access to ART or with delayed treatment initiation [31].

Over the past two decades, as ART has shifted the HIV population toward longer survival and preserved immune function, the pathogenic role of HCMV has expanded beyond classical AIDS-defining illnesses. HCMV has increasingly been implicated in non-AIDS-related conditions, a heterogeneous group of disorders, often inflammatory, cardiovascular, or metabolic in nature, that, while not defining AIDS, substantially contribute to long-term morbidity and mortality in individuals with chronic HIV infection [32,33] (Table 2). In pediatric populations, the clinical manifestations of HIV-associated vasculopathy and cardiometabolic dysfunction may emerge across a broad age range, from early infancy through late adolescence. Cerebrovascular abnormalities, including arteriopathy and stroke, have been reported even in young children with perinatally acquired HIV, whereas cardiometabolic alterations are more commonly detected during later childhood and adolescence, often as subclinical abnormalities identified through imaging or laboratory screening [32,33,34].

Multiple studies indicate that even in the absence of severe immunosuppression, HCMV coinfection is associated with persistent immune activation, accelerated HIV disease progression, and increased all-cause mortality [34,35,36]. Notably, an analysis of the ICONA cohort, comprising over 6000 adults living with HIV, demonstrated that HCMV seropositivity was associated with a 53% increased risk of developing a serious non-AIDS-defining event or dying from non-AIDS-related causes over 15 years of follow-up. While HCMV was not significantly associated with non-AIDS malignancies or non-vascular neurological disorders, it was independently linked to a 2.3-fold increased risk of cerebrovascular and cardiovascular disease [34].

The substantial contribution of HCMV to non-infectious, inflammation-driven comorbidities in adults suggests that analogous mechanisms may operate in pediatric populations, particularly among children living with HIV and those who are HEU.

### 3.2. HCMV Coinfection and Outcomes in Children Living with HIV

In children with advanced HIV-related immunosuppression, HCMV coinfection has been consistently associated with multiple AIDS-defining conditions, including pneumonia, retinitis, enteritis, hepatitis, encephalopathy, and esophagitis [7,37,38] (Table 2). One of the earliest studies from the United States, examining a cohort of ART-naïve pediatric patients, reported that HCMV coinfection conferred a 2.5-fold increased risk of death or progression to AIDS-defining illness. In addition, HCMV-positive children had a 2.9-fold higher risk of HIV-associated encephalopathy and a 2.5-fold increased likelihood of meeting CDC category C criteria, reflecting the most advanced stage of pediatric HIV disease [7]. Similar patterns were observed in other cohorts, in which symptomatic HCMV infection frequently coincided with early mortality during HIV coinfection [23,38].

In immunocompetent adults with HIV, plasma HCMV DNA is rarely detectable unless CD4^+^ T-cell counts fall below 200 cells/mm^3^ [39,40]. In contrast, infants living with HIV often exhibit prolonged and high-level HCMV viremia, frequently exceeding 1000 HCMV DNA copies/mL, which may persist for two years or longer and correlates with plasma HIV RNA levels [20]. Since early reports in the 1990s, plasma HCMV viral load has been recognized as an independent predictor of mortality in both adults and children living with HIV, and elevated HCMV DNA levels have also been strongly associated with the development of HCMV disease [38,41].

Consistent with these observations, a recent study of 163 children aged 2 months to 12 years who were hospitalized with newly diagnosed HIV infection, 78% of whom were severely immunosuppressed, found that 54% were HCMV-positive at admission, including 32% with HCMV viral loads ≥1000 IU/mL. Children in this high-viremia group had a significantly increased risk of death or prolonged hospitalization (>15 days), as well as higher 6-month mortality [42].

In contrast, a recent longitudinal study from Canada involving children living with HIV receiving ART reported no significant differences in short-term clinical outcomes between HCMV-positive and HCMV-negative participants. Although this finding appears discordant with earlier studies, it likely reflects the protective effect of early and sustained ART, which prevents severe immunosuppression and high-level HCMV viremia historically associated with excess morbidity and mortality. Nevertheless, HCMV-positive children in this cohort exhibited evidence of HCMV-driven immune remodeling, suggesting potential long-term consequences for chronic inflammation, immune aging, and non-AIDS-related comorbidities [43].

Taken together, available evidence indicates that the clinical impact of HCMV co-infection in children living with HIV is largely determined by the degree of immunosuppression and by HIV and HCMV viral loads at diagnosis. Importantly, cCMV remains the leading cause of sensorineural hearing loss and neurodevelopmental impairment in industrialized countries [44]. Accordingly, the high prevalence of cCMV among children living with HIV may further increase the risk of long-term auditory and neurological sequelae (Table 2). However, findings across cohorts are not entirely consistent; notably, in the PACTG 366 study, HCMV infection was not associated with neurocognitive performance as assessed by a comprehensive battery of standardized tests [45]. This discrepancy may reflect differences in study populations, timing of HCMV infection, the influence of antiretroviral therapy, as well as other confounding factors that influence neurocognitive development.

### 3.3. HCMV Infection and Outcomes in HIV-Exposed Uninfected Children

As the population of HEU children continues to grow because of widespread ART, it has become increasingly important to determine whether HCMV infection adversely affects health outcomes in this vulnerable group. As discussed above, rates of cCMV infection are higher in HEU infants than in HIV-unexposed children. Given that cCMV is a leading cause of sensorineural hearing loss (SNHL), a substantial proportion of HEU infants with cCMV infection are likely to develop severe hearing impairment [39,46]. In addition, HEU infants frequently exhibit higher and more prolonged HCMV viremia following primary infection, a feature that has been identified as a risk factor for hearing loss in some studies [20,44,47].

Beyond auditory outcomes, HCMV infection has been implicated in impaired growth and neurodevelopment among HEU children. A study of 120 Zambian children born to mothers living with HIV reported that postnatal HCMV infection was associated with reduced linear growth at 18 months of age, as well as smaller head circumference and lower scores on the Bayley psychomotor development scale [48]. However, disentangling the specific contribution of HCMV from the broader effects of HIV exposure and associated socioeconomic and environmental factors remains challenging.

Additional studies have yielded mixed results regarding growth outcomes. In Malawi, higher HCMV DNA concentrations in breast milk were associated with slower growth during the first six months of life among HEU infants [49]. In contrast, a separate cohort study found no association between HCMV viremia at 24 weeks of age and growth parameters [21]. Notably, in the same study, detection of HCMV DNA in infant plasma at 24 weeks was associated with an approximately four-fold increased risk of subsequent HIV acquisition, raising concern that HCMV infection may increase susceptibility to HIV and thereby indirectly contribute to adverse clinical outcomes related to HCMV–HIV coinfection (Table 2).

More recent evidence supports the notion that HCMV viral load is a critical determinant of clinical outcomes in HEU children. In a comparative analysis of HEU and HIV-unexposed children under five years of age, Pavlinac et al. reported that HEU children with HCMV viral loads ≥1000 IU/mL had a markedly increased risk of mortality within six months following hospital discharge (hazard ratio [HR] ~32.0) [50]. Moreover, each 1-log increase in HCMV viral load was associated with a significant increase in mortality risk (HR ~5.04). Importantly, this association was not observed in HIV-unexposed and uninfected (HUU) children, suggesting a unique vulnerability of HEU infants to the pathogenic effects of HCMV.

## 4. Cooperative Mechanisms of HCMV and HIV in Pathogenesis

A growing body of evidence indicates that HCMV and HIV interact through multiple direct and indirect mechanisms that mutually enhance viral replication, dissemination, and pathogenicity. Consistent correlations between HIV RNA and HCMV DNA viral loads have been observed in plasma, saliva, breast milk, and cervical secretions of coinfected individuals [20,39], underscoring the biological interplay between these two viruses across systemic and mucosal compartments.

Acute HCMV infection is characterized by robust activation and expansion of CD4^+^ T cells, which represent the primary targets for HIV. This expansion increases the pool of susceptible cells and may contribute to elevated HIV viral loads, even in individuals receiving ART [51]. In addition, HCMV and HIV share multiple cellular targets, including CD34+ progenitor cells, macrophages, and CD4^+^ and CD8^+^ T lymphocytes, facilitating viral interactions within common cellular reservoirs.

Several HCMV-encoded factors have been shown to directly promote HIV entry and spread. HCMV expresses Fc receptor–like proteins capable of binding HIV particles opsonized by non-neutralizing antibodies. This interaction facilitates antibody-dependent enhancement (ADE) of HIV infection by promoting viral uptake into target cells [52]. Another HCMV-encoded protein, pUS28, functions as a chemokine receptor that can substitute for the canonical HIV coreceptor CCR5, thereby promoting HIV entry and dissemination [53]. Moreover, HCMV infection itself induces upregulation of CCR5 expression, further enhancing HIV susceptibility [54].

More recently, mechanistic insights into mucosal coinfection have been provided by studies using polarized ex vivo tonsillar tissue explants, a relevant model for MTCT [55]. These studies demonstrated that HIV-1 and its proteins gp120 and Tat disrupt tight junctions within the tonsillar epithelium, increasing paracellular permeability and facilitating HCMV paracellular spread and epithelial infection. Subsequent dissemination of HCMV to macrophages and dendritic cells was observed. Conversely, HCMV infection of epithelial cells similarly compromised tight junction integrity, enhancing paracellular HIV-1 translocation across the mucosa and increasing viral access to CD4^+^ T cells, macrophages, and dendritic cells.

Persistent immune activation is a central feature of HIV pathogenesis and is further amplified by HCMV coinfection. HIV-induced dysregulation of cytokine networks results in elevated levels of pro-inflammatory mediators that drive chronic inflammation, immune exhaustion, and tissue damage [56]. HCMV infection similarly induces sustained overproduction of cytokines such as IFN-γ, TNF-α, and IL-6. Notably, HCMV-driven IL-6 overexpression has been independently associated with increased mortality in people living with HIV [57,58].

Consistent with these findings, individuals with HCMV–HIV coinfection exhibit significantly higher circulating levels of inflammatory markers, including IP-10 and TNF receptor II, compared with those infected with HIV alone [59,60,61]. This heightened inflammatory milieu is thought to contribute to the development of non-AIDS-related comorbidities, such as metabolic, cardiovascular, and renal diseases. Although these conditions have been insufficiently studied in pediatric HIV cohorts, they are expected to become increasingly relevant as life expectancy among children receiving ART continues to improve [62].

Finally, HCMV is well recognized for its profound immunomodulatory capacity, which enables lifelong persistence but may also induce durable alterations in immune homeostasis [63,64]. In the Swedish OCTO and NONA studies, HCMV infection was associated with an immune aging–like phenotype characterized by expansion of CD28^-^CD57^+^CD8^+^ T cells and a reduced CD4:CD8 ratio, features that predicted increased mortality and impaired cellular immune responses [65,66].

In the following chapter, we will examine in greater detail the phenomenon of immunosenescence induced by HIV and HCMV, with particular emphasis on how their synergistic effects may compromise immune development and long-term immune competence in pediatric populations.

## 5. Cellular Senescence, Immunosenescence, and Aging in HIV and HCMV Infections

### 5.1. Cellular Senescence and Immunosenescence

Cellular senescence was originally defined as a stable cell cycle arrest accompanied by resistance to apoptosis, serving as a protective mechanism to prevent malignant transformation of cells harboring irreparable DNA damage. However, subsequent studies have expanded this concept considerably, revealing that cellular senescence encompasses a broad spectrum of biological processes across multiple cell types [67,68].

Senescence can be considered a programmed cellular response to stressful events imposed by acute or chronic pressures. Acute senescence promotes tissue remodeling, ultimately leading to the clearance of senescent cells and restoration of homeostasis. In contrast, chronic senescence is a degenerative state, associated with age-related pathologies [69].

One of the most extensively characterized drivers of chronic senescence is telomere shortening resulting from the end-replication problem in somatic cells lacking sustained telomerase activity, in contrast to embryonic and stem-like cells [70,71,72]. The exposed ends of excessively shortened telomeres activate DNA damage response (DDR) pathways centered on the tumor suppressor p53, leading to the induction of cyclin-dependent kinase inhibitors such as p16 and p21 and resulting in irreversible G0/G1 cell-cycle arrest [68,73,74]. Concomitantly, senescent cells increase the production of interleukins, chemokines, growth factors, and proteases, defining the so-called Senescence-Associated Secretory Phenotype (SASP), which represents a central pathogenic feature of chronic senescence [67,75].

**Figure 1:**
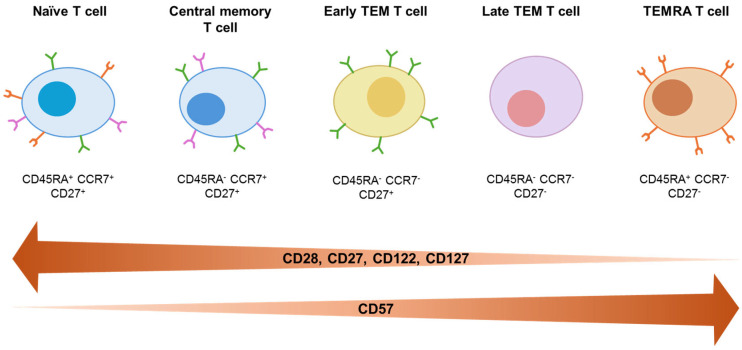
T cell maturation. CD45RA is shown in orange, CCR7 in pink, and CD27 in green.

Closely related to cellular senescence is the concept of immunosenescence, which refers to age- and antigen exposure-associated alterations in immune system composition and function. Its hallmarks include a decline in naïve T-cell numbers and T-cell receptor diversity, accumulation of terminally differentiated effector T cells, and chronic low-grade systemic inflammation [76]. These features are closely linked to cellular senescence, as telomerase activity is transiently upregulated via IL-7:IL-7R or IL-2:IL-2R interaction, to counteract telomere erosion associated with rapid cellular proliferation [77]. Similarly, antigen-stimulated memory T cells in the periphery can induce telomerase expression, but progressively declines with repeated proliferation and chronic antigenic stimulation [78,79,80]. Concomitantly, T cells exhibit reduced expression of co-stimulatory and cytokine receptor molecules, including CD28, CD27, CD122, and CD127, with loss of CD27 and CD28 closely associated with increased expression of the senescence marker CD57. Overall, in a linear differentiation model naïve T cells (CD45RA^+^CCR7^+^CD27^+^), upon first antigen encounter progressively differentiate into central memory (CM) T cells (CD45RA^-^CCR7^+^CD27^+^), CD27^+^ early effector memory (TEM) T cells, CD27^-^ late TEM T cells (CD45RA^-^CCR7^-^), and ultimately CD45RA re-expressing effector memory (TEMRA) T cells (CD45RA^+^CCR7^-^CD27^-^) (Figure 1) [81].

Overall, these observations support the existence of a direct mechanistic link between intense immune cell proliferation, telomere erosion, cellular senescence, and immunosenescence.

### 5.2. HIV and Immunosenescence

Since immune cells are the primary targets of HIV, it is not surprising that HIV has been consistently associated with the development of an immune-senescent phenotype [82,83,84]. Compared to adults, children have an immune system that is still undergoing maturation at the time of viral exposure, which makes them unable to develop an efficient immune response. Therefore, in this context, HIV infection has more complex consequences and contributes to more rapid disease progression [85]. Moreover, lifelong exposure to both persistent viral antigen and antiretroviral therapy from early childhood sustains chronic immune activation, thereby accelerating immune aging.

The main alteration of the immune phenotype in both adults and children living with HIV is the inversion of the CD4^+^/CD8^+^ T cells ratio, a marker closely associated with disease progression and immune dysfunction. Notably, approximately 66% of HIV-infected children treated with ART can reconstitute a normal CD4^+^/CD8^+^ ratio [86], a substantially higher rate than that observed in adults [87]. This difference likely reflects the greater thymic output characteristic of childhood, which supports more robust naïve T-cell replenishment [88]. Nevertheless, despite effective ART, multiple studies have documented a persistent depletion of naïve CD4^+^ and CD8^+^ T cells (CD45RA^+^CCR7^+^) in both pediatric and adult HIV infection [89,90,91,92].

**Figure 2:**
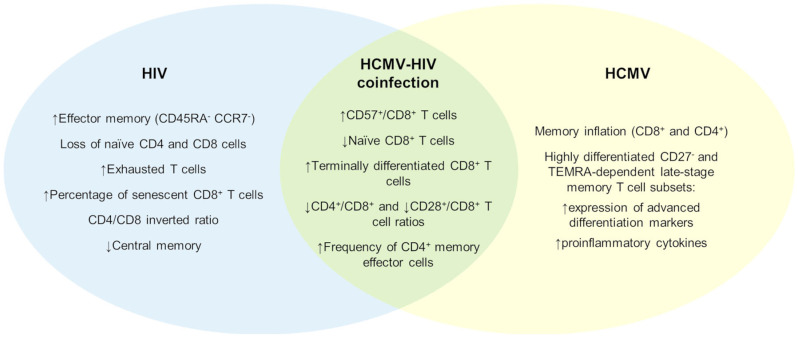
T cell landscape in HIV, HCMV, and HCMV–HIV coinfection (↑ increase; ↓ decrease of indicated factor).

In parallel, a reduction in central memory subsets and an expansion of effector memory T cells (CD45RA^-^CCR7^-^), with expansion of the CD27^-^ subset, has been detected [93]. Mansoor et al. directly compared HIV-infected infants, HEU infants, and HUU infants, demonstrating that HIV-infected infants exhibit profound and sustained alterations in T-cell composition throughout the first year of life (i.e., the entire follow-up). These included increased frequencies of memory, terminally differentiated (CD45RA^+^CCR7^-^), and senescent (CD28^-^CD57^+^) CD8^+^ effector T cells, accompanied by a marked reduction in naïve CD8^+^ T cells [94] (Figure 2).

Importantly, these immune alterations persist even under virological suppression. Several studies have shown that ART-treated children living with HIV retain elevated proportions of exhausted T cells, highlighting the close relationship between chronic immune activation, cellular exhaustion, and immunosenescence [95,96]. In line with this, Gianesin et al. demonstrated that children living with HIV accumulate activated, exhausted, and senescent CD8^+^ T cells, with these parameters inversely correlating with telomere length, providing direct evidence of accelerated biological aging [97]. Expression of PD-1, a key marker of HIV-associated T cell exhaustion, has also been proposed as a surrogate marker of disease progression [90].

Beyond T cells, HIV infection profoundly affects the B cell compartment. Multiple studies have shown that children living with HIV, including those with sustained viral suppression, exhibit B cell profiles resembling those of older healthy individuals [98,99]. These changes include an increased number of activated mature (CD10^-^CD21^-^) and senescent double-negative (IgD^-^CD27^-^) B cells. Chronic HIV persistence is also associated with expansion of immature transitional (CD10^+^/^++^CD21 low/high CD27^-^), activated memory (CD10^-^CD21 low CD27^+^), and exhausted memory (CD10^-^CD21 low CD27^-^) B cells, alongside a reduction in resting memory B cell subset (CD10^-^CD21 high CD27^+^) [100]. Furthermore, the expression of B cell genes that determine the response to vaccinations, including those involved in inflammation and aging, is altered even in the presence of stable and prolonged virological control [101].

### 5.3. HCMV and Immunosenescence

HCMV infection is known to be one of the most significant environmental drivers of immune system remodeling. Indeed, HCMV seropositivity has been associated with alterations in up to 50% of assessed immune parameters. The most marked changes are observed within T cells, particularly CD8^+^ T cells [102,103].

HCMV infection leads to a massive and atypical expansion of memory T cells, termed “memory inflation,” with up to 40% of all memory T cells targeting HCMV epitopes in infected individuals [102,103]. Although HCMV-specific CD4^+^ T cells also undergo expansion, this process is quantitatively less pronounced [104].

Inflated HCMV-specific T cells predominantly occupy highly differentiated late-stage memory subsets, including CD27^-^ effector memory and TEMRA populations. Phenotypically, HCMV-specific T cells display features of advanced differentiation, including high expression of CD57, KLRG1, CX3CR1, CD56, and NKG2C [105,106,107]. Both HCMV-specific CD8^+^ and CD4^+^ T cells also show a strong polyfunctionality, producing high levels of pro-inflammatory cytokines such as IFN-γ and TNF-α [108,109,110]. While most data on HCMV-driven immune remodeling derive from adult cohorts, similar alterations have also been documented in HCMV-infected children [105,106,107,111,112] (Figure 2).

Importantly, these alterations are not restricted to T lymphocytes. For example, children with cCMV infection exhibit marked alterations in NK cell composition compared to postnatally infected or uninfected controls [113]. In particular, expansion of CD94/NKG2C^+^ NK cells, a subset associated with adaptive-like features and immune aging, has been observed, especially in symptomatic congenital infections and in individuals with permissive NKG2C genotypes.

However, longitudinal studies tracking changes in blood immune cells shortly after infection and throughout the latent phase in children remain limited. However, a recent study by Ekman and colleagues analyzed children infected with HCMV before six months of age and followed them at six months and six years post-infection [114]. The authors observed that the most pronounced immune alterations occurred shortly after primary infection (at six months), affecting both the CD27^+^ early TEM and CD27^-^ late TEM/TEMRA in CD4^+^ and CD8^+^ subsets. Of note, the expansion of the most highly differentiated TEMRA subsets, expressing CD57 at high levels, persisted for at least six years, indicating long-lasting immune imprinting.

Intriguingly, emerging evidence suggests that HCMV may promote the accumulation of senescent cells not only within the immune system but also within infected tissue niches [115]. This may further contribute to premature aging and provides a novel mechanistic link between HCMV persistence, inflammaging, and long-term immune dysfunction.

### 5.4. HCMV–HIV Coinfection and “Premature” Aging

Despite the evidence discussed in the previous paragraphs, few studies have specifically analyzed the synergistic effects of HIV and HCMV infection on the immune system and premature aging. Most available data derive from adult cohorts, in which HCMV co-infection among people living with HIV has been consistently associated with immunosenescent immune profiles, although not necessarily with inflammation [116]. These observations suggest that HIV and HCMV may act synergistically to accelerate immune differentiation and exhaustion through partially overlapping but non-identical mechanisms (Figure 2).

Telomere erosion is markedly accelerated in individuals living with HIV, often exceeding that observed in significantly older uninfected controls. In this context, a recent study identified HCMV-specific CD8^+^ T cells as a population particularly prone to telomere shortening and rapid telomere attrition in people living with HIV [117]. Consistent with this, leukocyte telomere erosion in HIV infection has been linked to increased risks of cardiovascular disease, type 2 diabetes, malignancy, and other age-related comorbidities [118]. Notably, these associations mirror those described in elderly HCMV-seropositive but HIV-negative individuals, supporting the hypothesis that HCMV-driven clonal expansion is a major contributor to lymphocyte replicative senescence and age-related morbidity in the setting of HIV.

Evidence for accelerated immune aging in the context of HCMV–HIV coinfection is also emerging in pediatric populations. Children living with HIV coinfected with HCMV display higher frequencies of terminally differentiated CD8^+^ T cells and lower CD4^+^/CD8^+^ ratios compared with HCMV-negative peers. Importantly, these differences appear to be independent of detectable HCMV DNA or HIV RNA in peripheral blood, indicating that immune imprinting persists beyond the acute phase of either infection [119]. Similarly, Barrett et al. demonstrated that HCMV seropositivity in people living with HIV was associated with lower CD4^+^/CD8^+^ and CD28^+^/CD8^+^ T cell ratios, alongside increased frequencies of CD57^+^CD8^+^ T cells, independent of age and HIV viral load [120].

More recently, Fougère et al. provided a detailed immunophenotypic characterization of HCMV coinfection in children living with HIV (CLWH) in Canada [43]. Compared with HCMV-negative children, HCMV-exposed CLWH exhibited lower baseline frequencies of CD4^+^ T cells, higher frequencies of CD8^+^ T cells, and a persistently reduced CD4^+^/CD8^+^ ratio. In addition, HCMV-positive children showed an expansion of effector memory CD4^+^ and CD8^+^ T cell subsets, coupled with a contraction of the naïve CD8^+^ T cell pool, hallmarks of immune aging typically associated with much older individuals.

## 6. Conclusions

Collectively, the studies discussed in this review indicate that HCMV coinfection is common among children living with HIV and is associated with a distinct immunological signature, even in the presence of effective ART and sustained virological suppression.

Available evidence underscores the need for increased clinical and research attention to early HCMV–HIV coinfection in pediatric populations. From a clinical perspective, systematic screening for congenital and early postnatal HCMV infection in children living with HIV and in HEU infants should be considered, particularly in settings where maternal HIV infection is prevalent. Longitudinal monitoring of HCMV viral burden, immune activation markers, and early signs of neurodevelopmental, cardiovascular, and metabolic dysfunction may help identify children at increased risk for long-term morbidity. However, major knowledge gaps remain. These include the lack of prospective pediatric studies disentangling the independent and synergistic effects of HCMV and HIV on immune maturation, aging, and end-organ damage; limited data on the timing, reversibility, and clinical significance of HCMV-driven immune activation in the context of early and sustained ART; and the absence of validated biomarkers to guide risk stratification and targeted interventions. In addition, the potential role of anti-HCMV preventive or therapeutic strategies in improving long-term outcomes for coinfected children remains largely unexplored. Current evidence is largely derived from heterogeneous cohorts that often lack precise temporal classification of viral acquisition, standardized virological measurements, and longitudinal immune profiling. Addressing these gaps through well-designed longitudinal and interventional studies will be essential to inform evidence-based management strategies and optimize lifelong health outcomes in children affected by HCMV–HIV coinfection.

## Figures and Tables

**Table 1:** Diagnostic criteria for congenital vs. postnatal HCMV [15] and HIV [12,14] infection.

	HCMV	HIV
**Congenital infection**	▪ Positive PCR on saliva, urine, or blood within 21 days of life. Alternatively, positive PCR on dried blood spots (DBS) from neonatal screening ▪ Prenatal evidence includes: maternal seroconversion during pregnancy; detection of HCMV DNA by PCR in amniotic fluid	▪ Positive HIV RNA or DNA PCR on whole blood within 48 h of birth is highly suggestive of intrauterine infection
**Intrapartum infection**	▪ Not used as a distinct clinical category, as HCMV is not typically transmitted during childbirth	▪ Negative PCR on whole blood at 0–48 h, subsequently positive at 7–14 days of postnatal age
**Postnatal infection**	▪ Positive PCR on urine, saliva, or blood collected after 21 days of life (particularly in breastfed infants)	▪ Negative PCR on whole blood in the first weeks of life, subsequently positive several months later (typically in breastfed infants)
**Diagnostic notes**	▪ Serology is not useful in newborns ▪ If the first test is performed >21 days of life, congenital and postnatal infection cannot be reliably distinguished	▪ Antibodies are not interpretable before 18 months of age (reflect maternal status) ▪ Two positive PCR tests are required for confirmation

**Table 2:** AIDS-related vs. non-AIDS-related conditions in the pediatric population [32,33,34].

	Description	Examples	Role of HCMV Coinfection
**AIDS-related conditions**	AIDS-defining conditions due to severe immunodeficiency (markedly low CD4^+^ count)	▪ ***Opportunistic infections*** (HIV+): *Pneumocystis jirovecii* pneumonia, esophageal candidiasis, cerebral toxoplasmosis, HCMV retinitis ▪ ***AIDS-defining tumors***: aggressive lymphomas ▪ ***Other CDC category C conditions:*** HIV encephalopathy, recurrent severe bacterial sepsis	▪ Increased susceptibility and earlier onset of AIDS-related conditions ▪ Associated with a more rapid decline in CD4^+^ cell count ▪ Higher early mortality during the first year of life ▪ Congenital or perinatal HCMV infection is an independent risk factor for progression to AIDS
**Non–AIDS-related conditions**	Morbidities arising not from severe immunodeficiency but from chronic immune activation, inflammation, early HIV exposure, long-term antiretroviral therapy, and coinfections (common even with suppressed viremia)	▪ ***Growth dysfunctions*** (HIV+, HEU): height and weight retardation ▪ ***Neurocognitive dysfunctions*** (HIV+, HEU): psychomotor retardation, neurocognitive deficits ▪ ***Cardiometabolic dysfunctions*** (HIV+): dyslipidemia, atherosclerosis, insulin resistance ▪ ***Renal dysfunctions***: HIVAN (HIV-associated nephropathy) and other nephropathies ▪ ***Chronic pulmonary dysfunctions:*** bronchiectasis ▪ ***Immunological dysfunctions*** (HIV+): persistent immune activation, premature aging	▪ A strong driver of immune activation and systemic inflammation ▪ Increases the risk of neurocognitive delay, chronic lung disease, and sensorineural hearing loss ▪ Associated with endothelial dysfunction and early cardiovascular changes ▪ Contributes to long-term immune imprinting leading to early immunosenescence ▪ Increases vulnerability to common infections

## Data Availability

No new data were created or analyzed in this study.

## Abbreviations

The following abbreviations are used in this manuscript:

HCMV	Human cytomegalovirus
HIV	Human Immunodeficiency Virus
cCMV	Congenital HCMV
AIDS	Acquired Immunodeficiency Syndrome
MTCT	Mother-to-child transmission
ART	Antiretroviral therapy
HEU	HIV-exposed but uninfected
HUU	HIV-unexposed and uninfected
TEM	Effector Memory T cells
TEMRA	Terminally Differentiated Effector Memory T cells Re-expressing CD45RA
